# Behavioral Diversity as a Potential Indicator of Positive Animal Welfare

**DOI:** 10.3390/ani10071211

**Published:** 2020-07-16

**Authors:** Lance J. Miller, Greg A. Vicino, Jessica Sheftel, Lisa K. Lauderdale

**Affiliations:** 1 Chicago Zoological Society—Brookfield Zoo, Brookfield, IL 60513, USA; lisa.lauderdale@czs.org; 2 San Diego Zoo Global, San Diego, CA 92101, USA; gvicino@sandiegozoo.org (G.A.V.); jsheftel@sandiegozoo.org (J.S.)

**Keywords:** animal behavior, behavioral restriction, behavior-based enrichment, evidence-based management, fecal glucocorticoid metabolites, stereotypic behavior, thrive, welfare assessment

## Abstract

**Simple Summary:**

The zoological community continually looks for new ways to improve the management of the animals under their professional care. This includes finding opportunities for animals to thrive within a zoo or aquarium environment. Lack of negative indicators of welfare does not demonstrate that an individual animal is in a positive state. Thus, it is necessary to have positive indicators of animal welfare. There is a growing body of evidence that suggests that behavioral diversity may be a potential positive indicator of animal welfare. This review article highlights previous research on behavioral diversity, details the methods to calculate behavioral diversity, and provides guidance on how to use it for an evidence-based animal management program within a zoo or aquarium.

**Abstract:**

Modern day zoos and aquariums continuously assess the welfare of their animals and use evidence to make informed management decisions. Historically, many of the indicators of animal welfare used to assess the collection are negative indicators of welfare, such as stereotypic behavior. However, a lack of negative indicators of animal welfare does not demonstrate that an individual animal is thriving. There is a need for validated measures of positive animal welfare and there is a growing body of evidence that supports the use of behavioral diversity as a positive indicator of welfare. This includes an inverse relationship with stereotypic behavior as well as fecal glucocorticoid metabolites and is typically higher in situations thought to promote positive welfare. This review article highlights previous research on behavioral diversity as a potential positive indicator of welfare. Details are provided on how to calculate behavioral diversity and how to use it when evaluating animal welfare. Finally, the review will indicate how behavioral diversity can be used to inform an evidence-based management approach to animal care and welfare.

## 1. Introduction

Historically, the field of animal welfare focused on negative indicators of welfare [1]. This focus on the negative, instead of focusing on enhancement, resulted in the creation of minimum standards for animals [1]. An example includes the five freedoms that were created to help ensure that the welfare of animals was not compromised [2]. The freedoms were (1) freedom from hunger and thirst, (2) freedom from discomfort, (3) freedom from pain, injury, or disease, (4) freedom to express normal behavior, and (5) freedom from fear and distress [3]. However, the field continues to push forward and new frameworks for animal welfare have been developed focusing more on the positive. The Five Domains Model, originally designed to focus on experimental welfare compromise [4], was later modified to also focus on welfare enhancement [5]. Similarly, the Opportunities to Thrive were designed to allow for a focus on the empirical presence of positive indicators of animal welfare [6]. The opportunities include (1) opportunity for a thoughtfully presented, well-balanced diet, (2) opportunity to self-maintain, (3) opportunity for optimal health, (4) opportunity to express species-specific behavior, and (5) opportunity for choice and control. Supplementary Materials Document S1 details the Opportunities to Thrive [6], for the purpose of this manuscript, we will focus on the opportunity to express species-specific behavior.

In order to provide this opportunity, quality spaces and appropriate social groupings need to be provided that encourage species-specific behaviors at natural frequencies and of appropriate diversity while meeting the social and developmental needs of each species [6]. Historically, stereotypic behavior (or abnormal repetitive behavior) would have been examined to evaluate the welfare of individuals [1]. However, absence of negative indicators of welfare does not suggest that an individual animal is thriving. In order to assess the opportunity to express species-specific behavior, a potential positive indicator of welfare is behavioral diversity. Behavioral diversity can be defined as a measure of the richness of behavior (number of behaviors) as well as the evenness (frequency of each behavior) which aligns with the fourth opportunity. The theory behind behavioral diversity as a potential positive indicator of welfare is that when behavioral diversity is high, it is possible we are meeting the behavioral needs of an animal [7]. However, when behavioral diversity is low, an animal is likely stereotyping or lethargic, both of which are potential signs of compromised welfare [8].

The field of animal welfare is in need of additional positive indicators of animal welfare. While behavioral diversity has not been validated as a positive indicator of welfare, there is a growing body of evidence that would suggest it may be a useful tool in evaluating welfare. In addition, there has been a recent increase in scientists suggesting that behavioral diversity may be an important component of a welfare assessment or for assessing factors that impact welfare [9,10,11]. The following review will demonstrate the bigger picture of how behavioral diversity relates to multiple other indicators of welfare, including the identification of factors that have been found to increase or decrease behavioral diversity, detail methods to calculate behavioral diversity, and provide an overview of how behavioral diversity can be used for an evidence-based animal management program. The framework presented is one potential way to measure and manage animal welfare under professional care by outlining species-specific behaviors, contextualizing the behaviors, defining the natural history adaptations, defining successful outcomes, and determining inputs. The framework results in an outcome-based workflow for animal care professionals to follow that establish a list of species-specific, ecologically relevant experiences that can be used to promote behavioral diversity. The ultimate goals of the manuscript are to provide animal care professionals in zoos and aquariums a potential framework to explore and evaluate changes in behavioral diversity and increase scientific research evaluating the validity of behavioral diversity as a positive indicator of animal welfare.

## 2. Materials and Methods

The literature review was conducted by searching databases in Web of Science as well as Google Scholar. Search terms included behavioral diversity + welfare, behavioural (UK English) diversity + welfare, Shannon index + welfare, h index + welfare, behavior diversity + welfare, Shannon-Wiener + welfare, 1-simpson + welfare, and behavior diversity + zoo animal. The search resulted in 87 manuscripts that were read for suitability and searched for other potential references. An additional 28 manuscripts were identified to be reviewed for suitability, resulting in a total possible of 115 sources. The final sources for the review included articles in peer-reviewed publications as well as dissertations and are detailed in the reference section.

## 3. Behavioral Diversity and Behavioral Restriction

The idea that a higher behavioral diversity may be indicative of better animal welfare is based on current scientific understanding of behavioral restriction [12,13]. A number of studies have demonstrated that when animals have the inability to engage in certain behaviors they are motivated to perform, then welfare can be compromised (e.g., mink [14], pigs [15], horses [16], and mice [17]). If animals are engaged in a larger behavioral repertoire of species-appropriate behaviors, this would suggest that the likelihood of an individual not being able to perform a motivated behavior decreases with each additional behavior. This is not to suggest it eliminates that possibility, simply that the likelihood decreases with each additional behavior. Behavioral restriction is often a precursor to stereotypic behavior [18,19]. For example, parrots will develop stereotypic behavior when raised in barren environments [20]. When behavior is restricted, this typically leads to lower behavioral diversity and an increase in stereotypic behavior (e.g., sows [21]).

## 4. Behavioral Diversity and Stereotypic Behavior

Stereotypic behavior is not always directly related to compromised welfare, but should always be examined to understand the root cause [8]. Few studies have statistically examined the relationship between behavioral diversity and stereotypic behavior. While three studies have found a significant inverse relationship (Asiatic lion [22], New Zealand white rabbits [23], bottlenose dolphins [24]), a study on two bear species found no significant relationship [25]. Studies examining both behavioral diversity and stereotypic behavior but not comparing them statistically have generally found that when stereotypic behavior is high or increased, behavioral diversity is low or decreased (sows [21], small felids [26], large felids [27], giant pandas [28,29], spectacled bears [30], chimpanzees [31], African elephants [32], parakeets [33], and songbirds [34]). Similarly, pharmacologically induced stereotypic behavior was also associated with lower behavioral diversity [35].

There are some exceptions where a similar pattern was not observed between behavioral diversity and stereotypic behavior. Research on social housing in tamandua found pair housing to increase behavioral diversity and lower stereotypic behavior, however the latter was not significant [36]. Similarly, training of bottlenose dolphins for educational programs increased behavioral diversity but was unrelated to stereotypic behavior [37]. One potential reason stereotypic behavior was not impacted was due to overall low levels observed throughout the study. Similar results were also seen in a study examining enrichment with leopard geckos with stereotypic behavior not being impacted but was performed at a low rate [38]. Similar findings were also observed in a study examining enrichment in wombats with no impact on stereotypic behavior, but the authors attributed it to not providing the correct type of enrichment [39]. The final study that found concurrent results included examining enriching environments for capuchins with an increase in behavioral complexity and no impact on stereotypic behavior [40]. However, there have only been a few studies where both behaviors were examined when stereotypic behavior decreased or was lower and there was no impact on behavioral diversity. A study with lions found pacing was less frequent in a larger and less restrictive habitat but behavioral diversity was unaffected [41], and another study found enrichment decreased stereotypic behavior in squirrel monkeys but had no impact on behavioral diversity [42]. Finally, across 34 species of carnivores, behavioral diversity was higher for generalist species when compared to their specialist counterparts, but there was no relationship between diet in the wild and stereotypic behavior [43]. It is important to note that the way behavioral diversity is calculated across studies varies greatly, with some calculating indices while others have looked at activity budgets or the number of different behaviors observed. All of this should be considered when determining the validity of behavioral diversity as a measure of positive welfare.

## 5. Factors Affecting Behavioral Diversity

Behavioral diversity is often found to be higher following animal management practices thought to improve the welfare of animals. This includes environmental enrichment or habitat complexity, appropriate social groups, and animal training. The majority of the studies have examined the impact of enrichment on behavioral diversity as this was one of the original goals for enrichment programs [44]. In total, across 28 studies, there was a reported increase in behavioral diversity (decrease for lack of enrichment) for 78.6% of the studies, with the remaining 21.4% reporting no significant difference. Species that experienced an increase in behavioral diversity following enrichment or an enhanced habitat included big cats [27,45,46], leopard geckos [38], parakeets [33], capuchins [40], African cichlid males [47], pigs [21,48,49,50], wombats [39], red foxes [51], bottlenose dolphins [52], ghost bats [53], bears (spectacled [30], Andean, sloth, brown, and black [54]), rats [55], African elephants [32], small felids [26], hognose snakes [56], giant pandas [28,29], and chimpanzees [31]. Species where enrichment or improved habitat was not found to significantly change behavioral diversity include armadillos, bush babies, and two toed sloths [57], wolves [58], African elephants [59], zebra fish and checker barbs [60], and lions [41]. However, some of the species where enrichment did not increase behavioral diversity overlap with species were enrichment did increase behavioral diversity, so it could be the type or timing of the enrichment that is impacting the significance of results [61].

Studies examining other factors in relation to behavioral diversity are much more limited. As previously mentioned, training of bottlenose dolphins was found to significantly increase behavioral diversity [37]. Social housing and group living were found to relate to behavioral diversity in tamandua [36] and wolves [58]. Alternatively, social management was not found to relate to behavioral diversity in Asiatic lions [22].

While animal management factors that are thought to be important for welfare increase behavioral diversity, stressed or compromised animals tend to have lower behavioral diversity. Species infected with parasites or clinically impaired health tend to have lower behavioral diversity (Spanish ibex [62,63], Japanese macaques [64]). Shelter dogs also have lower behavioral diversity when first arriving at a shelter, likely a stressful situation, compared to four weeks later [65]. Even in the wild, chimpanzees negatively impacted by encroaching human populations demonstrated lower behavioral diversity [66]. While there are some exceptions, overall, factors thought to be positive from a welfare standpoint tend to be associated with higher levels of behavioral diversity and individuals that are stressed, clinically unhealthy, or restricted tend to have lower behavioral diversity.

## 6. Behavioral Diversity and Physiological Indicators of Animal Welfare

The final evidence that behavioral diversity may be a suitable positive indicator of animal welfare comes from studies examining relationships with physiological indicators of welfare. The earliest of these studies examined the relationship between behavioral diversity and fecal glucocorticoid metabolites in cheetahs. A significant inverse relationship was found between behavioral diversity calculated using Shannon’s diversity index [67] and fecal glucocorticoid metabolites [7]. Subsequent studies demonstrated a similar relationship in chimpanzees [68] as well as bottlenose dolphins [24]. Additionally, there was also an inverse relationship found between cortisol: dehydroepiandrosterone (DHEA) metabolite ratio and behavioral diversity for bottlenose dolphins [24]. While relatively new in the field of animal welfare, cortisol:DHEA is a marker used to examine chronic stress in humans with elevated ratios linked to adrenal fatigue, depression, or illness [69]. Given that cortisol can also rise in positive situations and under a variety of situations [70,71], this ratio may be a better indicator for comparison moving forward, however additional research is necessary to better understand the value of the ratio. The only study to our knowledge that has demonstrated a different relationship was with cheetahs [72]. In this study, there was a positive relationship between behavioral diversity and fecal glucocorticoid metabolites. However, rest was included in the calculation of behavioral diversity and may account for the differences observed between the two studies with cheetahs as it was described as a very commonly observed behavior in that study. However, it is also possible that the difference exists as one study looked at cheetahs in multiple locations compared to just one location. While clearly more research is necessary, previous research comparing behavioral diversity to physiological indicators of welfare provides further support as a positive indicator of animal welfare.

## 7. Methods of Calculation

Previous research has taken multiple approaches to quantifying the diversity of observed behaviors. Indices of diversity commonly used to describe the range of behaviors exhibited by animals include behavioral richness [37], behavioral evenness [59], behavioral variety index [42], activity budgets [27], Shannon’s diversity index [43], Shannon’s equitability [50], Gini-Simpson index [73], power spectral density [62], multifactorial correspondence analysis [58], and Zipf-Mandelbrot Law [40]. Table 1 presents the number of publications that specified utilizing a specific approach.

Diversity indices provide a method for assessing both the richness and evenness of a set of expressed behaviors. The set of behaviors included in the analysis are based on the behaviors defined in the ethogram used during data collection. Behavioral diversity is determined by the number of different behaviors from the ethogram that were expressed and the heterogeneity of their expression. Indices quantify diversity and are measures of uncertainty, not measures of diversity per se. However, the index values can be used as measures of equivalency. It is important to distinguish between the outcome units of each index and ensure they are not treated interchangeably in analyses and interpretation. Here, we summarize each of the indices utilized and detail the methods of calculation for some of the most common indices starting from least prevalent to most common in the literature.

Behavioral Evenness: Evenness represents the frequency or duration of each behavior type [74]. With this calculation of evenness, the variety of behaviors is maximized when all behaviors are expressed completely evenly. If all observed behaviors are exhibited equally, they would have a uniform distribution. If a few behaviors are dominant, then the behavioral repertoire is considered to be uneven. Evenness is an important component of diversity because a few incredibly dominant behaviors in some species may be an indication of a lack of behavioral flexibility or an environment that does not provide enough of an opportunity to engage in species-typical behaviors.

Gini-Simpson index: The Gini-Simpson index is a transformation of the original Simpson index. The Simpson index is a quantitative measure that also reflects the richness and evenness of behaviors [75]. It is equivalent to the Herfindahl index and the Herfindahl-Hirschman index in economics. This index is a weighted arithmetic mean of proportional abundance and measures the probability that two behaviors randomly selected from a sample will be the same. While the Simpson index is the probability that a randomly selected data point represents the same behavior, the Gini-Simpson equates to the probability that the two behaviors represent different behaviors [76]. The index is also known as the Gibbs-Martin index, the probability of interspecific encounter, expected heterozygosity, and the Blau index in other fields. Both the inverse Simpson and Gini-Simpson have been referred to as the simply the ‘Simpson index’ despite their transformations. Therefore, it is important for future studies to correctly identify the proper index in order to accurately interpret values.

Multifactorial Correspondence Analysis: Multiple correspondence analysis (MCA) is a type of principal component analysis for categorical data. To conduct an MCA, the correspondence analysis algorithm is applied to an indicated matrix. The analysis converts the matrix into a graphical format where the rows and columns of the matrix are graphed as points in geometric space [77]. The relative proximity of the points on the graph are indicative of their degree of correlation, with closer proximity representing higher correlation. The degree of similarity between variables is described by the χ^2^ criterion [78].

Zipf-Mandelbrot Law: The Zipf-Mandelbrot Law is utilized for measuring the complexity of behavioral sequences and is a reformulated Zipf’s law [79,80,81]. Zipf’s Law characterized the relationship between the frequency of a behavioral event within repertoire to its rank. Zipf’s Law states that a number of elements with a frequency is a random variable with power law distribution. Zipf-Mandelbrot Law is simplified version of Zipf’s equation and is also a power law distribution on ranked data.

Power Spectral Density: Power spectrum density (also called power spectrum) is a tool useful for characterizing the complexity of fractal behavior [82,83]. Data is analyzed by applying the Fast Fourier Transformation algorithm to time series binary data. Power spectral density shows changes in the spectral properties of time series data.

Behavioral Variety Index: The behavioral variety index is a frequency-based measure that quantifies the number of behavior types expressed by an animal. To calculate a behavioral variety index, individual behaviors are grouped into categories of related behaviors (e.g., exploration, social, locomotion [42]). Each behavior type within the group is coded as 0 for the absence of the behavior and 1 for the presence of the behavior. The coded behavior types are summed to create an index value for each category. A summary behavioral variety index can be calculated by summing the group indices.

Shannon’s Equitability: While Shannon’s diversity index is the most common index and will be explained in detail below, Shannon’s equitability (also called the relative index, Pielou’s evenness index, and the Shannon H/H max) can be calculated. Shannon’s equitability compares the estimated value of Shannon’s diversity index with a theoretically maximal value that could be obtained if the distribution were completely even [84].

Behavioral Richness: Behavioral richness (also referred to as behavioral counts) is frequency-based and the simplest diversity index [85]. Richness represents the count of the different behavior types exhibited by the animal in the corresponding ethogram. Behavioral richness (R) is calculated as:

(1) $$R=\overset{B}{\underset{i=1}{\sum }}n$$

where B is the number of behavior types and *n* is 1 if the behavior type occurred and zero if the behavior type did not occur. Richness is one of the most intuitive diversity indices because it is linear and follows the doubling property (e.g., a richness value twice as large indicates twice the diversity). However, behavioral richness is less informative and more imprecise than other diversity measures. It does not take into account the frequency with which the behaviors occur and is susceptible to more random variation.

Activity Budgets: An activity budget is the simplest diversity measure that includes both richness and evenness. As stated above, richness represents the number of behavior types observed. Evenness represents the frequency or duration of each behavior type. To create an activity budget, data can be collected using continuous or instantaneous methods. The duration or number of the observed behaviors are then summed [37]. The sum is then divided by the total time observed or total number of visible scans. The result is a percentage of time or proportion of visible scans that an animal is engaged in different behaviors. Activity budgets assist in developing a general picture of behavior and how much time an animal engages in various behaviors (i.e., foraging, locomotion, play).

Shannon’s Diversity Index: Shannon’s diversity index is the most commonly used index to describe behavioral diversity in animals (Table 1). It is also referred to as Shannon entropy, the Shannon-Wiener diversity index, and the Shannon-Weaver diversity index. This nonlinear index quantifies the uncertainty of selecting a specific behavior item on a given ethogram that is taken from a dataset [67,86]. Shannon’s diversity index (H) is calculated as:

(2) $$H=-\overset{B}{\underset{i=1}{\sum }}p_{i}\;ln\;p_{i}$$

where *B* is the number of behavior types and $p_{i}$ is the proportion of behavior i. An adaption of Shannon’s diversity index has also been used [36] and is calculated as:

(3) $$H=-\overset{B}{\underset{i=1}{\sum }}p_{i}\;log\;\left(\frac{1}{p_{i}}\right)$$

where *B* is the number of behavior types and $p_{i}$ is the proportion of behavior i. Index values increase with larger numbers of behavior types and more equal abundances. Shannon’s diversity index is one of the most accurate and useful diversity indices. Due to its nonlinear nature, it is less intuitive to interpret than linear measures such as behavioral richness. However, given the normal patterns of behavior observed in animals, the Shannon’s diversity index is ideal as it accounts for subtle changes in behavioral diversity even when one behavior (factor) is dominant [87].

## 8. Behavioral Diversity Considerations

While there is a growing body of evidence around behavioral diversity as a potential positive indicator of welfare, there are some factors to consider when utilizing the Shannon index to examine behavioral diversity [88]. While many of the points raised in Cronin and Ross [88] are based on hypothetical examples that do not represent normal behavioral patterns of real animals, they do have a few important points to consider. The concerns raised about the Shannon index could be seen as areas of opportunity for further research. First, when utilizing diversity indices, one is abiding by the assumptions that all behaviors are equally important (i.e., without regard to their functional role or valance). Additionally, it is important to point out that all behaviors must be equally detectable. The theory behind behavioral diversity is that for every additional behavior that is observed, there is a decreased likelihood that the animal is behaviorally restricted to perform a motivated behavior. Again, this does not eliminate the possibility of behavioral restriction, but decreases the likelihood. Research evaluating the inclusion and exclusion of different behaviors would be important to better understand the Shannon’s diversity index as a potential positive indicator of animal welfare. Second, diversity indices are also constrained by the number and/or grouping of behaviors into categories and, therefore, should only be directly compared when animals have been observed following the same ethogram. However, recent evidence suggests that the Shannon’s diversity index is fairly robust at measuring behavioral diversity as long as only species-specific behaviors are included in the calculation [68]. Further, as in all studies within the field of animal welfare, scientists should not solely rely on an index value and should examine the changes in a variety of indicators of welfare (e.g., other behaviors, physiology, etc.) in order to interpret the results and have a better understanding of where an animal might fall on a continuum of welfare. Again, additional research evaluating how these factors impact the Shannon’s diversity index would add value to its potential use as a positive indicator of animal welfare.

Additional considerations should be given to what behaviors to include for an ethogram exploring behavioral diversity. First, given the theory behind behavioral diversity as a positive indicator of welfare, it would be recommended to only utilize species-typical behaviors [7,24,68]. While including behaviors such as rest (lethargy) or stereotypic behavior on an ethogram is important for exploring the entire continuum of animal welfare, it would be recommended to not include these behaviors in behavioral diversity calculations. If behavioral diversity represents the likelihood that we are meeting the behavioral needs of an individual animal or species, then only utilizing species-typical behaviors would be appropriate. It appears that the Shannon’s diversity index is fairly robust as an indicator of positive animal welfare [68], however additional research is necessary across a diverse taxonomic representation to better understand its value in the field of animal welfare.

## 9. Shannon’s Behavioral Diversity Index Scores across Species

As previously noted, the Shannon’s diversity index has been the most widely used index when examining behavioral diversity. While there are clearly more studies needed, previous research provides an idea of how scores vary across species and taxonomic groups. In order to better understand how behavioral diversity ranges across and within species, taxonomic groups, and management strategies, it is important for studies examining behavioral diversity to provide those values. Unfortunately, in many of the studies reviewed, actual values and ranges were not provided and estimates cannot be made from graphs. It is recommended that all future studies examining behavioral diversity provide those values either in text or table format for future reference and comparison across studies. Table 2 provides a summary of index values using the Shannon’s diversity index.

## 10. Application of Behavioral Diversity

With a clear understanding of how to effectively calculate behavioral diversity, one potential applied use of the index can be a multi-phase process to measure and manage animal welfare under professional care. The first requirement is a range of behaviors observed from a wild or managed population of the target species robust enough to evaluate the current behavioral repertoire of a species or individual. Using the Opportunities to Thrive model described in the introduction, the measurement phase begins by asking true or false questions about the observed behavioral outcomes expressed by the target individual or species:

1. Does the animal express behavior at a frequency and diversity that is consistent with natural history?
2. Does the animal express normal social behavior?
3. Does the animal respond in a behaviorally appropriate way to challenges, problem solving, and environmental changes in a way that is consistent with natural history?
4. Is the animal responsive to learning new skills and shows motivation to engage?
5. Does the animal use the environment to acquire resources that benefit them?

After determining which of the behavioral groupings have (or have not) met an acceptable standard, a husbandry (management) plan can be developed using the following outcome-based behavior workflow. The tool is a systematic approach that allows practitioners to determine what behaviors are meaningful to the target species based on natural history and then develop experiences that will elicit those behaviors. To begin, it is critical to have a strong understanding of the individual animals’ behaviors and the subsequent relevance to their ecology.

Step 1. Using the template (Table 3), begin by outlining species-specific behaviors. These behaviors can include anything one would expect to see in the wild, as well as any individual behaviors exhibited under professional care. For this example, we have arbitrarily limited the number of initial behaviors to five, but this column will be informed by the known repertoire of the species in question.

Step 2. Breakdown each behavior into its contexts and/or components by listing circumstances where a species may choose to execute the behavior. In this example, the context of the behavior “Hunting” is broken down into different types of prey that may be available to a wild counterpart. The behavior “Hunting” could also be broken down into the components of the behavior itself (for example: seek, stalk, pounce, process).

Step 3. List the natural history adaptations that allows the specific species to execute the behavior according to the context and/or components. For this step, describe how those contexts are relevant to the animals’ adaptations and natural history. Practitioners must think about how those contexts differentiate how the animals’ adaptations allow it to cope with those differences.

Step 4. Determine what outcomes could be measured if successful at getting the animal or species to elicit the behavior. In this step, one should use the previous categories of context and/or components and natural history adaptations to describe exactly how the context dictates the use of particular adaptations to accomplish the behavior. Essentially, this is what it looks like for the animal to accomplish the behavior using its adaptations to do so, in the context that it is presented. These behavioral outcomes must be measurable and can be incorporated into the subsequent measurement of changes to behavioral diversity.

Step 5. Describe the inputs used to achieve the behavioral goals of replicating the adaptively relevant aspects of a snow leopard “hunting”. The term input is simply a place holder, and can refer to a practice, structure, or technique that is added or employed to achieve the desired outcome. The input(s) should incorporate knowledge and thought from all previous categories to form a rich and complex experience.

The outcome-based behavior workflow results in a list of experiences for the species that not only is ecologically relevant to the species natural history, but also allows the evaluation of its implementations by measuring behavioral diversity through the process. By focusing on behavioral diversity instead of novel objects, resources can be re-directed from a traditional object-based husbandry program to experiences that are truly meaningful to the species. These experiences can be coupled or layered, and can unfold over the course of several days, with a relevant and unique set of cues, preceding or following them. For example, if it rains, one can initiate a weeklong experience (“hunting rodents”) that only happens following a rain event, thus strengthening the relationship between the animal and the natural world. Behavioral diversity is the foundation of the layered implementation of inputs, resulting in meaningful species-specific experiences.

From the perspective of a practical and applied approach, a behavioral diversity index can be used to measure the efficacy of the above programmatic change in husbandry. Using the Shannon’s diversity index, one should also include other behaviors of interest to ensure a balanced assessment. For example, if we are interested in reducing pacing, it is important to not only monitor that specific behavior, but also behavioral diversity to better understand how an intervention is impacting the overall behavior and welfare of an individual or species. Behavioral data were collected on an Amur leopard between November and December of 2017 (pre) and again in November and December of 2018 (post), following the implementation of an outcome-based husbandry program. Designed to be approachable to practitioners, the programmatic shift includes abandoning the traditional input- or occupational-based enrichment program and replacing it with a husbandry program that is based on experiences developed by the outcome-based behavioral workflow. For instance, feeding paradigms no longer follow a schedule based on the animal care professional, rather they are all components of a temporally extended (sometimes up to five days) series of adaptively relevant cues indicating the ultimate presence of an opportunity for large prey, cached carrion, large amounts of small prey, etc. Twenty minute continuous focal observations were conducted for all occurrence behavior using ZooMonitor four times a day twice a week, balanced between two morning observations (7:00–12:00) and two afternoon observations (12:00–18:00), using a simplified ethogram to increase usability and reliability (Table 4.)

**Figure 1:**
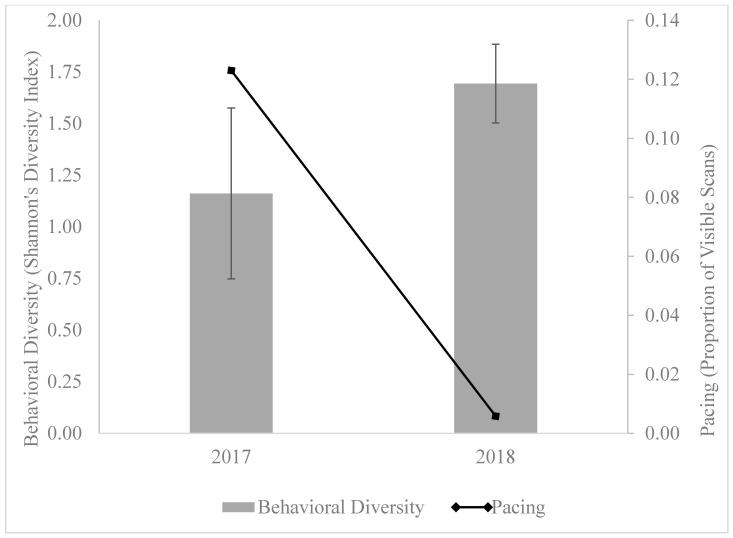
Behavioral diversity and pacing behavior of Amur leopard before and after utilizing outcome-based behavior workflow.

Pre and post Shannon’s diversity index values from a single Amur leopard before and after a fundamental shift in husbandry informed by an outcome-based behavior workflow are presented in Figure 1. In this example, the diversity index increases, while the proportion of pacing behavior decreases, suggesting a successful intervention. The increase in behavioral diversity and the reduction of pacing behavior allows practitioners to visualize the value of a more diverse behavioral repertoire and the potential relationship between increased diversity and decreased stereotypic behavior. This basic assessment can be applied anytime the outcome-based behavior workflow is utilized to try and improve the behavioral diversity of a species under professional care.

## 11. Conclusions and Future Directions

The main goals of the current paper were to provide animal care professionals at zoos and aquariums a framework to explore and evaluate changes in behavioral diversity and to encourage scientists to continue to examine and validate behavioral diversity as a potential positive indicator of welfare. The framework presented a multi-phase process to measure and manage animal welfare under professional care by outlining species-specific behaviors, contextualizing the behaviors, defining the natural history adaptations, defining successful outcomes, and determining inputs. The framework results in an outcome-based behavior workflow for animal care professionals to follow that establish a list of species-specific, ecologically relevant experiences that can be used to promote behavioral diversity. The reviewed literature suggests that behavioral diversity may be a useful tool in evaluating welfare and that further work needs to be done to fully understand behavioral diversity within the context of animal welfare.

In terms of examining the validity of behavioral diversity as a positive indicator of welfare, future studies could examine the relationship with other behavioral and physiological measures. This could include the relationship between behavioral diversity and indicators of welfare such as stereotypic behavior, lethargy, immunoglobulin A (IgA), or cortisol:DHEA ratio. In addition, as already outlined, there is a need to examine the impact of the grouping of behaviors in ethograms on behavioral diversity. More broadly, research is also needed to define normal and/or ideal diversity values for a variety of species across taxonomic groups and determine if there is value in the ability to compare index values across species.

An additional area of opportunity includes determining if the Shannon’s diversity index is the best index moving forward. The article outlines many of the indices used historically, and there are other indices yet to be used within the field of animal welfare. For example, transforming Shannon’s diversity index and Simpson diversity index into true diversity values has been employed in other fields [86,91,92,93]. Converting these numbers to true diversities (i.e., the effective number of behaviors) may provide benefits regarding interpretation. Future research should explore the possible usefulness of these transformations.

There is still considerable work needed to validate behavioral diversity as an indicator of positive welfare and develop behavioral diversity reference values across more taxonomic groups. Despite the continued efforts necessary to fully understand behavioral diversity, we recommend moving forward with goals to increase the diversity of ecologically relevant, desirable behaviors in an applied setting. While unlikely based on current evidence, if future research deems that behavioral diversity is not a positive indicator of animal welfare, there are still many reasons to focus on behavioral diversity. Research has demonstrated that visitors to zoological institutions report feeling more emotionally connected to wildlife when they observe animals engaging in a variety of species-appropriate behavior [94,95]. In addition, we doubt that having a high behavioral diversity of species-appropriate behaviors would compromise the welfare of individual animals. We hope this manuscript encourages staff at zoos and aquariums to focus on increasing behavioral diversity within species-appropriate behaviors, and for scientists to continue to examine behavioral diversity as a potential positive indicator of welfare.

## Figures and Tables

**Table 1:** Number of publications that include each calculation method for behavioral diversity.

Calculation Method	Number of Publications
Shannon’s Diversity Index	28
Activity Budgets	9
Behavioral Richness	7
Shannon’s Equitability	4
Behavioral Variety Index	2
Power Spectral Density	2
Behavioral Evenness	1
Gini-Simpson Index	1
Multifactorial Correspondence Analysis	1
Zipf-Mandelbrot Law	1

**Table 2:** Shannon’s diversity index scores across species.

Species	Shannon’s Diversity Index Range	Reference
Ghost Bats	3.22–3.69	[53]
African Elephants	1.43–2.11	[59]
Domestic Dogs	0.92–1.20	[65]
Asiatic Lion	0.73–1.26	[22]
Black and Sun Bears	0.69–2.09	[25]
Tamandua	0.53–2.89	[36]
Leopard Cats	0.46–0.55	[26]
Leopard Gecko	0.41–0.75	[38]
Fishing Cats	0.31–0.55	[26]
Gentoo Penguins	0.28–1.74	[89]
Carnivores (34 Species)	0.20–2.01	[43]
Hognose Snake	0.10–0.43	[56]
Cheetah	0.03–0.20	[7]

**Table 3:** Sample outcome-based behavior workflow for snow leopard hunting behavior *.

**Step 1.** Identify species-specific behaviors	Play	Drink	Scent Mark	Hunt			
**Step 2.** Identify contexts or components	-	-	-	Small-Sized Prey	Medium-Sized Prey	Large-Sized Prey	Birds
**Step 3.** List the natural history adaptations	-	-	-	Quick reaction time, quick response to unpredictable spatial or temporal appearance. Uses multiple sensory cues. High risk of failure.	Relies on sensory cues. Must use claws, legs, teeth, eyes, ears, balance, muscles, speed, and strength. Risk of failure or injury.	Relies on sensory cues, seasonal cues, and must use claws, legs, teeth, eyes, ears, balance, muscles, speed, and strength. Risk of failure or injury.	Stalking, noise reduction, vertical leaping ability, long leaping ability, and paw eye coordination. Uses multiple sensory cues.
**Step 4.** Determine behavioral outcomes	-	-	-	Limited window of opportunity, pouncing, improved accuracy, opportunistic, and fast responses. High energy output for small reward.	Use correct responses to cues over a long period of time. Problem solving, stalking, physically demanding, and unpredictable. High value and high motivation.	Long buildup of responses to sensory and seasonal cues, stalking, jumping, and long pre-process time (attack/kill), stashing, dragging heavy objects, high motivation, high reward, and high energy output.	Must jump and swipe, unpredictable, must be fast and have quick reaction time, and vertical pursuits. High output for small reward.
**Step 5.** Identify inputs to achieve behavioral goals	-	-	-	Offer smaller, whole prey items in hard to reach places. Only available for a designated period of time. Attached to zip line that retreats into an inaccessible area. Rabbit in polyvinyl chloride (PVC) pipe in the ground (with or without bungee). Preceded by automatic scent dispenser, environmental changes (holes dug, rabbit sounds, bushes shaking, and small pieces of pelt) for days prior.	Following scent, visual, and auditory cues for a period of days, food is located out of reach in novel locations and/or attached to bungee or other device. Offer medium-sized whole prey items following environmental events (rain, cloud cover, heat). Increase time allowed with food item, placed in a way that only some parts can be stashed if not removed by the cat properly.	Very rare following long period of visual cues (vultures), then scent cues, and finally auditory cues (other predators/scavengers, antler rubs, mating calls). Large and rare whole prey item. Very difficult to move, fixed at several points in a very difficult area to access. Only provided on occasion following a specific seasonal event (dry to wet).	Novel food item with timed delivery and quick dispersal. Small whole prey item like a bone on a vertical pulley, swipe tube, or cat operated zip line. Preceded with auditory cues days prior, and feathers closer to provision. Seasonally directed with high concentration time points after natural event (cold snap, rain event, or season change).

* Different colors of shadow mean different workflows for snow leopard hunting behavior.

**Table 4:** Ethogram of Amur leopard behavior. Adapted from Stanton et al. [90].

Behavior	Description
Pacing *	Walking in fixed pattern with little or no variation. Must complete 3 repetitions to qualify
Sit	Hind legs and rump in contact with surface
Stand	Upright position, all four paws on surface
Rest Alert *	Lying down, head up, alert, and responsive to stimulus
Rest Non-Alert *	Lying down, head down, and eyes mostly closed, generally immobile or sleeping
Flehman/Sniff	Flehman-Lips pulled back, mouth open, tongue partially extended to allow air flow. Sniff-inhaling air through nose in short repetitive manner, directed at the air, object or substrate
Scent Mark	Deposits odor on object(s) with intent. Includes scratch, rub, scrape, spray, and roll
Vocalize	Makes any vocalization
Self-Maintenance	Licking body or paw and passing the paw over the head, may include chewing on fur, squatting with elimination of feces or urine
Eat	Actively ingesting food item or water
Food Acquisition	Any obvious effort required to obtain food reward. Includes stalk, pounce, swat, claw
Play	Physically or visually engaged with an object, self (chasing paws or own tail) or conspecific in a non-serious manner
Social	Actively engaged in behavior with others. Same space—grooming, play, aggression, breeding, attack, pounce, stalk, and physical contact. Adjacent Space—Any obvious interaction/interest within one body length of another cat in an adjacent space. Allogrooming—grooming or being groomed by a conspecific
Locomotion	Forward motion, non-stereotypic
Out of View *	Out of view of the observer
Other *	Any other behavior not described above

* Not included in behavioral diversity calculation.

## Supplementary Materials

The following are available online at https://www.mdpi.com/2076-2615/10/7/1211/s1: Document S1: Opportunities to Thrive.
