# Correction: Miller et al. Behavioral Diversity as a Potential Indicator of Positive Animal Welfare. *Animals* 2020, *10*, 1211

**DOI:** 10.3390/ani13121904

**Published:** 2023-06-07

**Authors:** Lance J. Miller, Greg A. Vicino, Jessica Sheftel, Lisa K. Lauderdale

**Affiliations:** 1 Chicago Zoological Society—Brookfield Zoo, Brookfield, IL 60513, USA; lisa.lauderdale@czs.org; 2 San Diego Zoo Global, San Diego, CA 92101, USA; gvicino@sandiegozoo.org (G.A.V.); jsheftel@sandiegozoo.org (J.S.)

## Missing Citation

In the original publication [1], the source of the behavioral variety index was cited to the incorrect paper in two locations. The citation has been corrected to “Spiezio, C.; Valsecchi, V.; Sandri, C.; Regaiolli, B. Investigating individual and social behaviour of the Northern bald ibis (*Geronticus eremita*): Behavioural variety and welfare. *PeerJ* **2018**, *6*, e5436” [2]. The citation has now been inserted into the Section 7. Methods of Calculation; Paragraphs 1 and 8 and should read:

Paragraph 1: Previous research has taken multiple approaches to quantifying the diversity of observed behaviors. Indices of diversity commonly used to describe the range of behaviors exhibited by animals include behavioral richness [37], behavioral evenness [59], behavioral variety index [73], activity budgets [27], Shannon’s diversity index [43], Shannon’s equitability [50], Gini–Simpson index [74], power spectral density [62], multifactorial correspondence analysis [58], and Zipf–Mandelbrot Law [40]. Table 1 presents the number of publications that specified utilizing a specific approach.

Paragraph 8: Behavioral Variety Index: The behavioral variety index is a frequency-based measure that quantifies the number of behavior types expressed by an animal. To calculate a behavioral variety index, individual behaviors are grouped into categories of related behaviors (e.g., exploration, social, locomotion [73]). Each behavior type within the group is coded as 0 for the absence of the behavior and 1 for the presence of the behavior. The coded behavior types are summed to create an index value for each category. A summary behavioral variety index can be calculated by summing the group indices.

With this correction, the order of some references has been adjusted accordingly. The authors state that the scientific conclusions are unaffected. This correction was approved by the Academic Editor. The original publication has also been updated.
