# Supplementary material for: Behavioral Diversity as a Potential Indicator of Positive Animal Welfare
Source: Animals (Basel). 2020 Jul 16;10(7):1211. doi: 10.3390/ani10071211 (PMC7401597; doi:10.3390/ani10071211)
Supplement: Supplementary file 1 [file animals-10-01211-s001.pdf]

Opportunities to Thrive [6]

1. Opportunity for a thoughtfully presented, well-balanced diet: A suitable, species-specific diet will be provided in a way that ensures full health and vigor, both behaviorally and physically.
2. Opportunity to self-maintain: An appropriate environment including shelter and species-specific substrates that encourage opportunities to self-maintain.
3. Opportunity for optimal health: Rapid diagnosis and treatment of injury or disease while providing supportive environments that increase the likelihood of healthy individuals.
4. Opportunity to express species-specific behavior: Quality spaces and appropriate social groupings will be provided that encourage species-specific behaviors at natural frequencies and of appropriate diversity while meeting social and developmental needs of each species in the collection.
5. Opportunities for choice and control: Providing conditions in which animals can exercise control and make choices to avoid suffering and distress, and make behavior meaningful.
